# Scalp Acupuncture and Treadmill Training Inhibits Neuronal Apoptosis through Activating cIAP1 in Cerebral Ischemia Rats

**DOI:** 10.1155/2021/1418616

**Published:** 2021-11-12

**Authors:** Qiang Tang, Tao Ye, Runyu Liang, Yan Wang, Hongyu Li, Jiyao Zhang, Xingxing Yuan, Luwen Zhu

**Affiliations:** ^1^The Second Hospital, Heilongjiang University of Chinese Medicine, Harbin, Heilongjiang, China; ^2^First Affiliated Hospital of Guizhou University of Traditional Chinese Medicine, Guiyang, Guizhou, China; ^3^Heilongjiang University of Chinese Medicine, Harbin, Heilongjiang, China; ^4^Heilongjiang Academy of Chinese Medicine, Harbin, Heilongjiang, China

## Abstract

Stroke is the leading cause of long-term disability in developed countries. Multitudinous evidence suggests that treadmill training treatment is beneficial for balance and stroke rehabilitation; however, the need for stroke therapy remains unmet. In the present study, a cerebral ischemia rat model was established by permanent middle cerebral artery occlusion (pMCAO) to explore the therapeutic effect and mechanism of scalp acupuncture combined with treadmill training on ischemic stroke. Terminal deoxynucleotidyl transferase-mediated dUTP nick-end labeling and neuronal nuclear protein (NeuN) double staining and cellular inhibitor of apoptosis protein-1 (cIAP1) and NeuN immunofluorescence double staining were used to detect the short-term and long-term neuroprotective effects of scalp acupuncture combined with treadmill training on pMCAO rats. In addition, the antiapoptotic effect of the combined treatment was evaluated in pMCAO rats transfected with cIAP1 shRNA. Western blotting was used to detect the relative protein expression in the caspase-8/-9/-3 activation pathway downstream of cIAP1 to further clarify its regulatory mechanism. Our results showed that scalp acupuncture combined with treadmill training successfully achieved short-term and long-term functional improvement within 14 days after stroke, significantly inhibited neuronal apoptosis, and upregulated the expression of cIAP1 protein in the ischemic penumbra area of the ischemic brain. However, no significant functional improvement and antiapoptotic effect were found in pMCAO rats transfected with cIAP1 shRNA. Western blotting results showed that the combined therapy markedly inhibited the activation of the caspase-8/-9/-3 pathway. These findings indicate that scalp acupuncture combined with treadmill training therapy may serve as a more effective alternative modality in the treatment of ischemic stroke, playing an antiapoptotic role by upregulating the expression of cIAP1 and inhibiting the activation of the caspase-8/-9/-3 pathway.

## 1. Introduction

Stroke is the leading cause of long-term disability in developed countries and is one of the top causes of mortality worldwide. China accounts for almost one-third of the total stroke deaths in the world; furthermore, over recent years, stroke has been the leading cause of dementia [1]. Ischemic stroke, defined as an acute focal injury of the central nervous system (CNS) which occurs when a blood vessel in the neck or brain is blocked, is one of the most common types of stroke, accounting for approximately 80% of all stroke incidents [2]. Ischemia and hypoxia of brain tissue further result in corresponding neurological deficit symptoms and signs due to brain infarction, necrosis, and the loss of a large number of neurons [3]. Changes in standing ability, sensorimotor function, and postural swing are the main poststroke complications and are closely related to the increased risk of falls and balance disorders [4, 5]. Therefore, balance is often the main focus of interventions in stroke rehabilitation.

Acupuncture is recommended by the World Health Organization as an alternative and complementary strategy. It is defined as the insertion of fine needles or sometimes a finger on the defined points and has been used for patients with stroke and poststroke rehabilitation for thousands of years [6, 7]. Recent research indicates that manual acupuncture or electroacupuncture activates afferent fibers at particular acupoints and then sends signals to the CNS, playing a role in improving balance, reducing spasms, increasing muscle strength, and general well-being poststroke via molecules identified as possible mediators of specific acupuncture effects, such as neurotransmitters (catecholamine, acetylcholine, 5-hydroxytryptamine, glutamate, and gamma-aminobutyric acid), neuropeptides, cytokines, and growth factors [7–9]. Scalp acupuncture is one of the several specialized acupuncture techniques in which a filiform needle is used to penetrate specific stimulation areas on the scalp, mainly for the treatment of brain diseases [10]. Systematic evidence has suggested that Baihui (GV20)-based scalp acupuncture is beneficial in the treatment of ischemic stroke as it improves infarct volume and neurological function scores and exerts a potential neuroprotective role in experimental ischemic stroke [11, 12]. However, the mechanisms underlying the beneficial effects of acupuncture in stroke rehabilitation remain unclear.

Poststroke rehabilitation training, such as treadmill training, has been shown to be effective in addressing the inefficiencies and gait patterns of poststroke individuals, principally through improving endurance and gait speed [13]. Despite the large number of studies conducted, the potential effects of treadmill training on balance function and postural control in poststroke individuals need to be further improved. In the present study, we hypothesized that scalp acupuncture combined with treadmill training therapy could effectively reduce nerve injury and promote the recovery of limb disorders. The neuronal expression level of cellular inhibitor of apoptosis protein-1 (cIAP1), which is considered the key target of neuroprotection induced by ischemic preconditioning, was examined *in vivo* to further investigate the regulatory effects of scalp acupuncture combined with treadmill training therapy in inhibiting neuronal apoptosis and neuroprotection.

## 2. Materials and Methods

### 2.1. Animals

The experiment was approved by the Animal Care and Use Committee of Heilongjiang University of Chinese Medicine (ABX20190508A) and executed following the National Institutes of Health Guidelines. Ten- to twelve-week-old male Sprague-Dawley rats (250 ± 14 g) were acquired from Liaoning Changsheng Biotechnology Co., Ltd. (SCXK 2015–0001) (Liaoning, China) and housed in a clean room with *ad libitum* access to food and water at a room temperature of 23–25°C and 55–65% humidity.

### 2.2. Development of cIAP1 shRNA

Three shRNAs, each targeting different regions of the rat *cIAP1* gene, and a negative control (NC) shRNA were designed and synthesized by Gene Pharma Co. (Shanghai, China). The target sequences were as follows: shRNA-1 : TTCTAGAG CAGTGGAAGAC, shRNA-2 : CTACAGGACGGTCAGTGAT, and shRNA-3 : CAG CTGCGGAGATTACAAG. shRNA-NC was a negative sequence with no significant homology to any rat gene.

### 2.3. Cell Culture and Transient Transfection

SH-SY5Y cells were obtained from the cell bank of the Chinese Academy of Science and were seeded into 25 cm^2^ flasks with DMEM medium (Sigma-Aldrich, MO, USA) containing 10% FBS and incubated in a 37°C, 5% CO_2_ thermostatic incubator. Cells were grown to confluency and passaged with 0.25% trypsin at 2–3-day intervals.

The cell concentration was adjusted to 1 × 10^6^ cells/ml and seeded in six-well plates. When the density of the cell slide reached 90%, they were transfected into five groups as follows: (1) control group, (2) negative group (shRNA-NC), (3) shRNA-1 group, (4) shRNA-2 group, and (5) shRNA-3 group. Transfection was performed according to the manufacturer's protocol. Transfection efficiency was observed by real-time PCR and Western blot at 48 h, and *cIAP1* shRNAs were screened to select the one with the highest interference efficiency for animal experiments.

### 2.4. Animal Study

The ischemic stroke model was established by permanent middle cerebral artery occlusion (pMCAO) as previously described [14]. Briefly, rats were anesthetized by an intraperitoneal injection of pentobarbital (45 mg/kg body weight). Next, a silicone-coated nylon monofilament was inserted through a small incision in the right common carotid artery and was then advanced to 18 mm distal to the carotid bifurcation through the internal carotid artery to occlude the origin of the middle cerebral artery. In sham-operated animals, the same procedure was performed with the exception of inserting the intraluminal filament.

One hundred and forty male Sprague-Dawley rats were randomly divided into the following five groups: (1) sham operation group (**S-con**), *n* = 28, sham operation without intervention; (2) model group (**M-con**), *n* = 28, pMCAO without intervention; (3) scalp acupuncture group (**SA**), *n* = 28, pMCAO with scalp acupuncture at the Baihui point (GV20) and 2 mm on the left and right sides. Cluster needling was carried out with a 0.25 × 25 mm acupuncture needle; the depth of needling was 15 mm, and the needle was kept for 2 h after 30 min of scalp electroacupuncture, once daily. The scalp electroacupuncture frequency was 2–15 Hz, and the stimulation intensity was 1 mA, induced by a G6805-2A instrument [15]. The fourth group was the treadmill training group (**TT**), *n* = 28, pMCAO with TT. An electric running platform was used for training: slope: 0°; speed: 8 m/min during day 1 to day 3, 12 m/min during day 4 to day 7, 15 m/min after day 7, 30 min per time, once daily. The fifth group was scalp acupuncture combined with treadmill training group (**SA-T**), *n* = 28, pMCAO with SA and TT, once daily.

**Figure 1:**
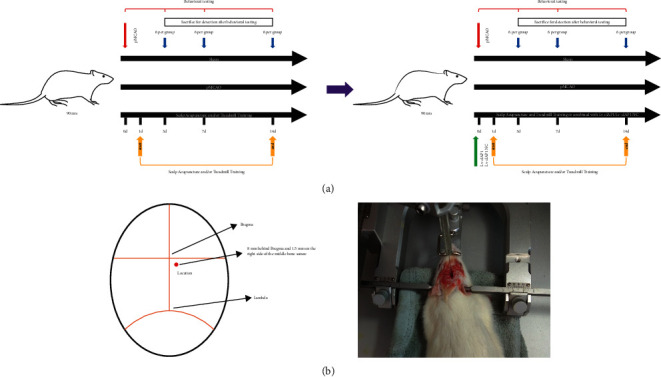
Schematic description of the study design and experimental operation. (a) Study design and experimental timeline. (b) Schematic diagram and operation diagram of the injection site of Lv-cIAP1.

In addition, 140 male Sprague-Dawley rats were randomly divided into the following five groups: (1) **S-con**, *n* = 28, sham operation with no intervention; (2) **M-con**, *n* = 28, pMCAO with no intervention; (3) **SA-T**, *n* = 28, pMCAO with SA and TT, once daily; (4) *cIAP1* negative control group (**SA-T** **+** **Lv-cIAP1 NC**), *n* = 28, in addition to SA and TT treatment, a lentiviral vector (1 × 10^9^ TU/ml, 2 *μ*l) negative control (Lv-*cIAP1* NC) was injected into the lateral ventricle 30 min before pMCAO with a stereotaxic instrument after the mice were anesthetized; and (5) the *cIAP1* interference group (**SA-T** **+** **Lv-cIAP1**), *n* = 28, in addition to SA and TT treatment, a lentiviral vector (1 × 10^9^ TU/ml, 2 *μ*l) interfering with *cIAP1* (Lv-cIAP1 NC) was injected into the lateral ventricle 30 min before pMCAO with a stereotaxic instrument after the rats were anesthetized. The injection was completed with a 10 *μ*L microsyringe at the rate of 0.2 *μ*L/min. A detailed schematic description of the study design and injection site of Lv-*cIAP1* is shown in Figure 1.

### 2.5. Behavioral Outcome Evaluation

The behavioral outcomes of rats were evaluated using the modified Bederson test [16], Beam Walk test [17], and Rota-rod test [18] before and 3, 7, and 14 days after modeling.

### 2.6. Histopathology

Brain tissue was embedded in paraffin, and then, consecutive cross-sections of the brain were cut. Hematoxylin-eosin (HE) staining and Nissl staining were performed to observe the neuronal damage in the ischemic penumbra on the 3rd, 7th, and 14th day after modeling.

### 2.7. Double Immunofluorescence

For terminal deoxynucleotidyl transferase-mediated dUTP nick-end labeling (TUNEL) and neuronal nuclear protein (NeuN) double staining, paraffin sections of the brain tissue on the 3rd, 7th, and 14th day after modeling were dewaxed and rehydrated by xylene and graded alcohol and then incubated at 37°C in ethylenediaminetetraacetic acid (EDTA) (pH 9.0) for 15 minutes. A TUNEL reaction mixture (Beyotime, China) was added and then blocked with 3% goat serum albumin for 30 min. The sections were then incubated with primary antibody against NeuN (0.72 *μ*g/ml, 24307, CST) at 4°C overnight and Cy3-labeled goat anti-rabbit IgG antibody (0.8 *μ*g/ml, ab6939, Abcam) for 50 min sequentially.

For cIAP1 and NeuN double staining, paraffin sections of the brain tissue on the 3rd, 7th, and 14th day after modeling were dewaxed and rehydrated by xylene and graded alcohol and then incubated in 37°C EDTA (pH 9.0) for 15 minutes. The sections were then incubated with primary antibody against NeuN (0.72 *μ*g/ml, 24307, CST) and cIAP1 (5 *μ*g/ml, ab25939, Abcam) at 4°C overnight. Then, Cy3-labeled goat anti-rabbit IgG antibody (0.8 *μ*g/ml, ab6939, Abcam) was added and incubated for 50 minutes sequentially. Finally, the sections were incubated with DAPI for 10 min prior to visualizing them with a fluorescence microscope (BX51, Olympus, Japan) at 400 magnification.

### 2.8. TUNEL Assay

The TUNEL assay was performed using the colorimetric TUNEL apoptosis assay kit (Beyotime, China) according to the manufacturer's instructions. Paraffin sections of the brain tissue on the 3rd, 7th, and 14th day after modeling were dewaxed and rehydrated by xylene and graded alcohol and then incubated at room temperature 3% H_2_O_2_ for 10 minutes. The sections were rinsed and visualized using 3,3′-diaminobenzidine (DAB), followed by the TUNEL reaction mixture. Cell nuclei were counterstained with hematoxylin. Visualization was carried out with an optical microscope (CX41, Olympus, Japan) at 400 magnification. Quantitative analysis was carried out by calculating the number of positive staining cells in the ischemic penumbra.

### 2.9. Immunohistochemistry Staining for cIAP1

The brain sections were then incubated with a 3% goat serum albumin dissolved in PBS at room temperature for 2 h to remove peroxidase. Next, the sections were incubated with primary antibody against cIAP1 (10 *μ*g/mL, ab25939, Abcam) at 4°C overnight, followed by incubation with a secondary antibody conjugated with HRP (2 *μ*g/mL, ab6721, Abcam) at 37°C for 45 min. Specific labeling was visualized with a DAB kit and appeared tan-colored, while cell nuclei were counterstained with hematoxylin. The visualization was carried out with an optical microscope (CX41, Olympus, Japan) at 400 magnification. Quantitative analysis was carried out by calculating the number of positive staining cells in the ischemic penumbra.

### 2.10. Western Blot Analysis

Proteins obtained from the SH-SY5Y cells or ischemic penumbra tissue of rats were run by 10% sodium dodecyl sulfate-polyacrylamide gel electrophoresis and then transferred onto a polyvinylidene difluoride membrane. Penumbral tissues were separated according to a previous study [19]. The brain was sectioned into three slices beginning 3 mm from the anterior tip of the frontal lobe. Regions from the right and left hemispheres of Section 2 that corresponded to the ischemic core and penumbra were dissected. We initially identified the midline between the two hemispheres and then made a longitudinal cut (from top to bottom) approximately 2 mm from the midline through each hemisphere. We then made a transverse diagonal cut at approximately the “2 o'clock” position to separate the core (i.e., striatum and overlying cortex) from the penumbra (adjacent cortex). Blots were blocked with 5% skimmed milk for 2 h and then incubated with primary antibodies against cIAP1 (2 *μ*g/mL, ab25939, Abcam), cleaved caspase-9 (0.09 *μ*g/mL, 20750, CST), cleaved caspase-8 (0.133 *μ*g/mL, 9496, CST), cleaved caspase-3 (0.0519 *μ*g/mL, 9661, CST), NeuN (0.036 *μ*g/mL, 24307, CST), and *β*-actin (0.061 *μ*g/mL, 4970, CST) at 4°C overnight. The following day, blots were incubated with HRP-labeled goat anti-rabbit IgG antibody (1 *μ*g/mL, ab6721, Abcam) for 2 h with shaking. Proteins were detected with enhanced chemiluminescence, and images were taken and examined with a Gel-Pro-Analyzer 4.0 (Media Cybernetics, USA).

### 2.11. Real-Time Polymerase Chain Reaction

Total RNA was isolated from SH-SY5Y cells using TRIzol reagent (Thermo Fisher, CA, USA) according to the manufacturer's instructions. RNA samples were reverse transcribed into cDNA with the PrimeScript^TM^ RT reagent kit (Takara, Kyoto, Japan). The mRNA expression of target genes was examined by a standard SYBR-Green method in the ABI PRISM 7500 sequence detection system (Applied Biosystems, CA, USA). The reaction conditions were as follows: predenaturation at 93°C for 3 min, 40 cycles of denaturation at 93°C for 30 sec, annealing at 55°C for 45 sec, and extension at 72°C for 45 sec. All results were normalized to the expression levels of *β-actin* and quantified by the comparative (2^−△△Ct^) method. The primer sequences were synthesized by Sangon Biotech (Shanghai, China) as follows: *cIAP1* forward 5′- GCAGGCTTCTACTACACAGGG-3′, reverse 5′-TTGGATACACTAAACCTCTGCG -3′; *β-actin* forward 5′-GGAGATTACTGCCCTGGCTCCTAGC-3′, reverse 5′-GGCCGGACTCATCGTACTCCTGCTT-3′.

### 2.12. Statistical Analysis

Experimental results are shown as mean ± standard error of the mean. Data were analyzed using SPSS 15.0 software. For analyses with only one factor involved, a one-way analysis of variance (ANOVA) and multiple comparisons followed by Bonferroni tests were applied, while a two-way ANOVA and multiple comparisons followed by Tukey's test were used when two factors were involved. All statistical tests were two-sided and *P* values <0.05 were considered statistically significant.

## 3. Results

### 3.1. Effect of SA-T on the Behavioral Outcomes of the pMCAO Model

**Figure 2:**
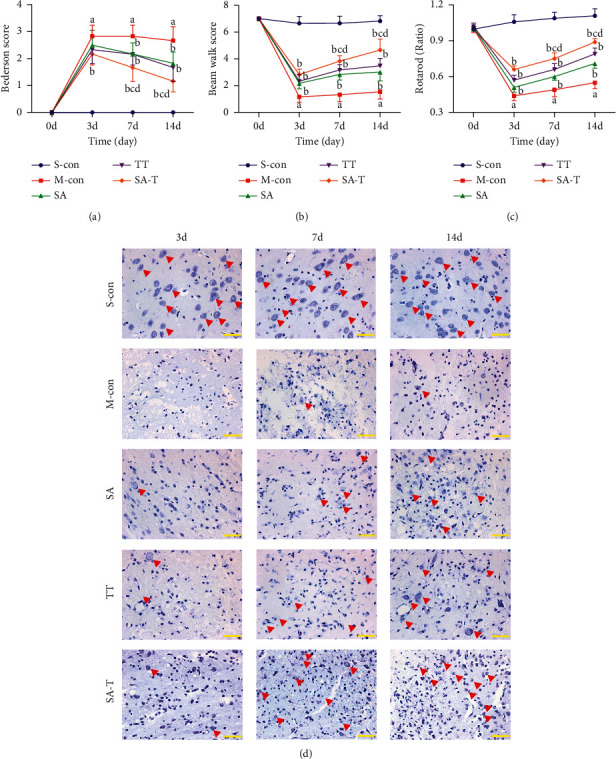
Effect of SA-T on behavioral outcomes and neuronal damage of the pMCAO model. (a), (b), (c) Behavioral outcomes were evaluated using a modified Bederson test, Beam Walk test, and Rota-rod test before and 3, 7, and 14 days upon pMCAO. (d) The brain tissues were stained with cresyl violet and observed using a microscope on the 3rd, 7th, and 14th days upon pMCAO (× 400). The red arrows mark intact neurons. Data are presented as mean ± SEM; *n* = 6 per group at each time point. ^a^*P* < 0.05 vs. S-con group, ^b^*P* < 0.05 vs. M-con group, ^c^*P* < 0.05 vs. SA group, ^d^*P* < 0.05 vs. TT group.

The behavioral results showed that SA-T successfully achieved short-term and long-term functional improvements within 14 days after stroke. The difference was statistically significant when compared with the model group (*P* < 0.05). Nevertheless, it should be noted that the difference in these improvements was significant in the SA-T group when compared with the other treatment groups on each day during the evaluation (*P* < 0.05). These results suggest that SA-T has a therapeutic effect on neurological deficits and is superior to SA and TT treatments (Figures 2(a)–2(c)).

### 3.2. Effect of SA-T on Neuronal Damage in the pMCAO Model

The Nissl body is sensitive to ischemic injury and appeared to decrease or disappear once ischemia occurred. Regarding the neuropathological changes in the cortex penumbra of the ischemic brain, the Nissl staining results showed a significant reduction in neurons of the model group when compared with the sham operation group on the 3rd, 7th, and 14th days after modeling. After treatment with SA, TT, or SA-T, increased Nissl bodies were observed indicating that these treatments were effective for ischemic stroke. It is noteworthy that SA-T was more neuroprotective than SA and TT (Figure 2(d)).

### 3.3. Effect of SA-T on Neuronal Apoptosis in the pMCAO Model

**Figure 3:**
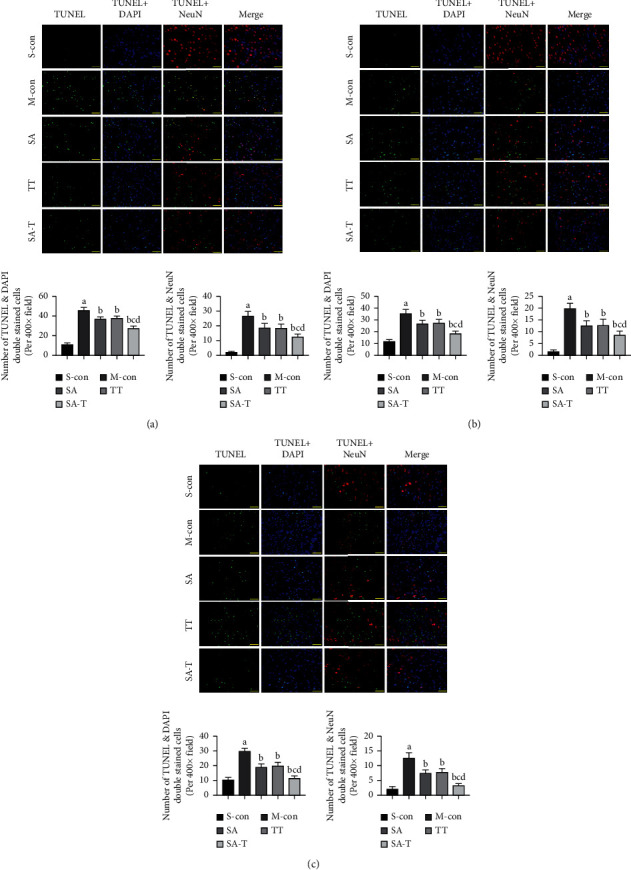
Effect of SA-T on neuronal apoptosis of the pMCAO model. (a) Quantification of TUNEL positive cells and NeuN and TUNEL double-positive cells on the 3rd day. (b) Quantification of TUNEL positive cells and NeuN and TUNEL double-positive cells on the 7th day. (c) Quantification of TUNEL positive cells and NeuN and TUNEL double-positive cells on the 14th day. Double immunofluorescence staining for TUNEL (green), NeuN (red), and nuclei-stained with DAPI (blue). Fluorescence images were captured using a fluorescence microscope (× 400). Data are presented as mean ± SEM; *n* = 6 per group at each time point. ^a^*P* < 0.05 vs. S-con group, ^b^*P* < 0.05 vs. M-con group, ^c^*P* < 0.05 vs. SA group, ^d^*P* < 0.05 vs. TT group.

NeuN is a specific protein expressed in the nucleus and cytoplasm of most neurons in the nervous system. We further examined the effect of SA-T on neuronal apoptosis in the ischemic penumbra area by double immunofluorescence staining (Figure 3). Limited numbers of TUNEL and NeuN positive neurons were observed in the sham operation group, while markedly more TUNEL and NeuN positive neurons were found in the model group on the 3rd, 7th, and 14th days (*P* < 0.05). SA, TT, and SA-T treatments significantly decreased the number of TUNEL and NeuN positive neurons in pMCAO rats (*P* < 0.05), and SA-T showed the highest antiapoptotic effect.

### 3.4. Effect of SA-T on cIAP1 Expression in Neurons of the pMCAO Model

**Figure 4:**
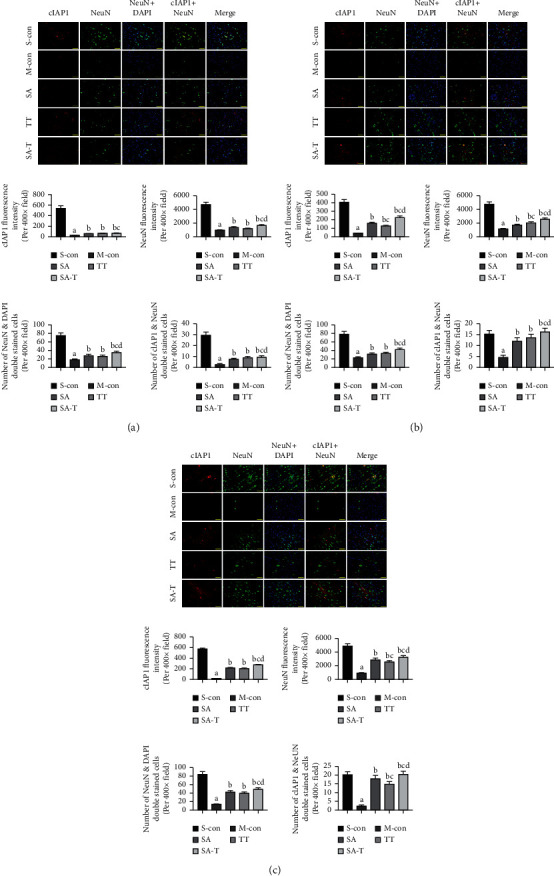
Effect of SA-T on cIAP1 expression in neurons of the pMCAO model on the 3rd day. (a) Quantification of fluorescence intensity of cIAP1 and NeuN and quantification of NeuN positive cells and cIAP1 and NeuN double-positive cells on the 3rd day. (b) Quantification of fluorescence intensity of cIAP1 and NeuN and quantification of NeuN positive cells and cIAP1 and NeuN double-positive cells on the 7th day. (c) Quantification of fluorescence intensity of cIAP1 and NeuN and quantification of NeuN positive cells and cIAP1 and NeuN double-positive cells on the 14th day. Double immunofluorescence staining for cIAP1 (red), NeuN (green), and nuclei-stained with DAPI (blue). Fluorescence images were captured using a fluorescence microscope (× 400). Data are presented as mean ± SEM; *n* = 6 per group at each time point. ^a^*P* < 0.05 vs. S-con group, ^b^*P* < 0.05 vs. M-con group, ^c^*P* < 0.05 vs. SA group, ^d^*P* < 0.05 vs. TT group.

Apoptosis is a critical factor for the loss of hypoxic-ischemic neurons in the nervous system, and cIAP1 is an important regulator of apoptosis [20, 21]. The double immunofluorescence staining results showed that multitudinous cIAP1 and NeuN positive neurons existed in the cortex penumbra of the ischemic brain in the sham-operated group (Figure 4). Compared with the sham-operated group, the fluorescence intensity of cIAP1 and NeuN proteins and the number of cIAP1 and NeuN positive neurons were markedly reduced on the 3rd, 7th, and 14th days in the model group (*P* < 0.05). SA, TT, and SA-T significantly increased the fluorescence intensity of cIAP1, NeuN, and the number of cIAP1 and NeuN positive neurons in pMCAO rats (*P* < 0.05), with SA-T showing the highest effect.

### 3.5. Effects of Three Pairs of shRNA Expression Vectors on cIAP1 Expression

**Figure 5:**
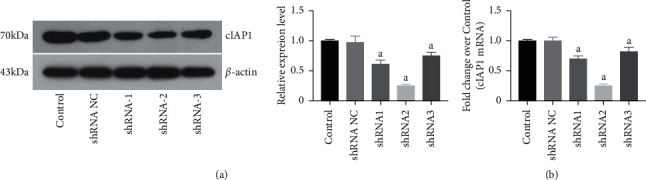
Effect of three pairs of shRNA expression vectors on cIAP1 protein expression. (a) Quantification of cIAP1 protein expression in SH-SY5Y cells. (b) Quantification of cIAP1 mRNA expression in SH-SY5Y cells. Data are presented as mean ± SEM; *n* = 3 per group. ^a^*P* < 0.05 vs. control group, ^b^*P* < 0.05 vs. shRNA-NC group.

The results of Western blotting and RT-PCR showed that there was no decline in cIAP1 protein or mRNA expression in the negative group (NC) as compared with the control group. However, the expression of cIAP1 protein was decreased by 39%, 75%, and 25% in the shRNA-1, shRNA-2, and shRNA-3 groups, respectively (Figure 5(a)). Moreover, *cIAP1* mRNA expression was decreased in the shRNA-1, shRNA-2, and shRNA-3 groups by ∼30%, 75%, and 18%, respectively (Figure 5(b)). These results suggest that shRNA-2 provided the most reliable expression. Therefore, shRNA-2 was selected for the subsequent experiments.

### 3.6. Effect of SA-T on Behavioral Outcomes in the cIAP1 shRNA Interference pMCAO Model

**Figure 6:**
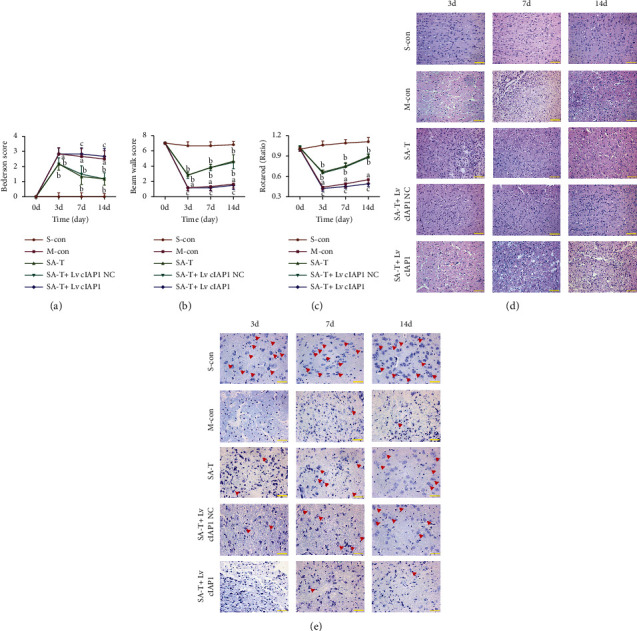
Effect of SA-T on behavioral outcomes in the cIAP1 shRNA interference pMCAO model. (a), (b), (c) Behavioral outcomes were evaluated using the modified Bederson test, Beam Walk test, and Rota-rod test before MCAO on the 3rd, 7^th^, and 14th day in the cIAP1 shRNA interference pMCAO model. (d) The brain tissues were stained with H&E and observed using a microscope on the 3rd, 7^th^, and 14th day in the cIAP1 shRNA interference pMCAO model (×200). (e) The brain tissues were stained with cresyl violet and observed using a microscope on the 3rd, 7^th^, and 14th day in the cIAP1 shRNA interference pMCAO model (× 400). Data are presented as mean ± SEM; *n* = 6 per group at each time point. ^a^*P* < 0.05 vs. S-con group, ^b^*P* < 0.05 vs. M-con group, ^c^*P* < 0.05 vs. SA-T group.

As shown in Figures 6(a)–6(c), the SA-T + Lv *cIAP1* NC group showed the same trend as SA-T in behavioral outcomes, and there was no difference between the two groups (*P* > 0.05). However, the therapeutic effect of SA-T on cerebral ischemia was completely reversed by Lv *cIAP1*. There was no significant difference between SA-T + Lv *cIAP1* and model groups (*P* > 0.05), and behavioral outcomes in the SA-T + Lv *cIAP1* group were significantly worse than in the SA-T group (*P* < 0.05).

### 3.7. Effect of SA-T on Neuronal Damage in the cIAP1 shRNA Interference pMCAO Model

HE and Nissl staining were conducted to evaluate the histological damage in the brain. The results of HE staining showed that most cells were arranged disorderly, and neuronal necrosis was observed in the model group. SA-T and SA-T + Lv *cIAP1* NC treatment markedly ameliorated these pathological abnormalities, but no significant difference was observed between the two groups (*P* > 0.05). Compared with the SA-T group, more neuronal necrosis existed in the SA-T + Lv *cIAP1* group, and the difference was statistically significant (*P* < 0.05) (Figure 6(d)). Nissl staining showed a similar trend with HE staining; fewer Nissl bodies were observed in the SA-T + Lv *cIAP1* group when compared with the SA-T group (*P* < 0.05) (Figure 6(e)).

### 3.8. Effect of SA-T on Neuronal Apoptosis and NeuN Expression in the cIAP1 shRNA Interference pMCAO Model

**Figure 7:**
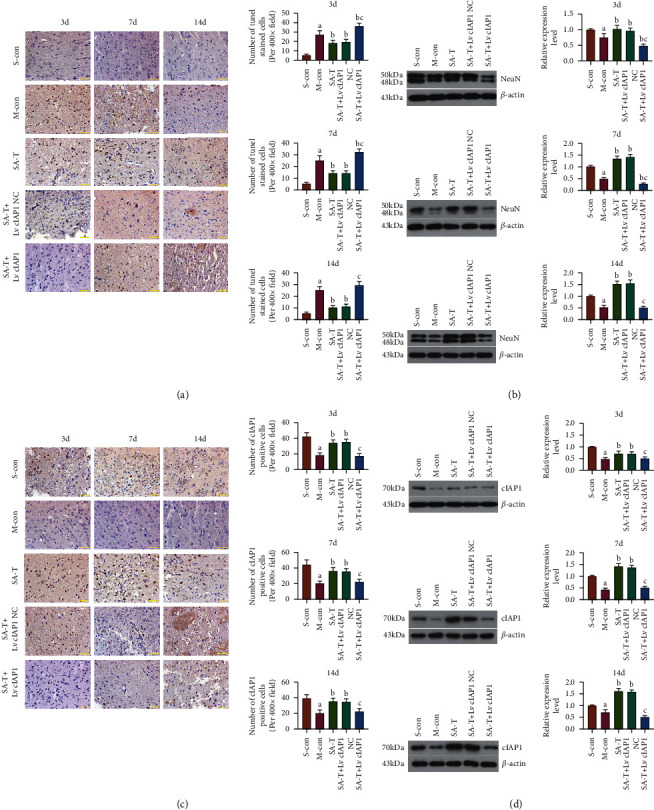
Effect of SA-T on neuronal apoptosis and the expression of NeuN and cIAP1 on the cIAP1 shRNA interference pMCAO model. (a) Quantification of TUNEL positive cells on the 3rd, 7^th^, and 14th days. Neuronal apoptosis using TUNEL assay. Images were captured using an optical microscope (× 400). (b) Detection of the expression level of NeuN protein in the brain by Western blot on the 3rd, 7^th^, and 14th days. (c) Quantification of cIAP1 positive cells on the 3rd, 7^th^, and 14th days. (d) Detection of the expression level of cIAP1 protein in the brain by Western blot on the 3rd, 7^th^, and 14th days. Data are presented as mean ± SEM; *n* = 6 per group at each time point. ^a^*P* < 0.05 vs. S-con group, ^b^*P* < 0.05 vs. M-con group, ^c^*P* < 0.05 vs. SA-T group.

Neuronal apoptosis in the *cIAP1* shRNA interference pMCAO model was further determined by a TUNEL assay. Treatment with SA-T alone or combined therapy with Lv *cIAP1* NC partly diminished neuronal apoptosis induced by cerebral ischemia (Figure 7(a)). The same results were also confirmed by Western blotting, which showed that the expression level of NeuN protein in the SA-T and SA-T + Lv *cIAP1* NC group significantly increased when compared with the model group (*P* < 0.05) (Figure 7(b)). However, in the SA-T + Lv *cIAP1* group, SA-T showed no significant antiapoptotic effect, and the number of TUNEL positive cells was markedly higher than that in the SA-T group (*P* < 0.05). In addition, Western blotting results showed that the NeuN protein expression level in the SA-T + Lv *cIAP1* group was significantly lower than that in the SA-T group (*P* < 0.05). Even on the 3rd and 7th days, the expression of NeuN protein in the SA-T + Lv *cIAP1* group was significantly lower than that in the model group (*P* < 0.05).

### 3.9. Effect of SA-T on cIAP1 Expression in the cIAP1 shRNA Interference pMCAO Model

To further investigate the influence of SA-T on the expression patterns of cIAP1 in the cIAP1 shRNA interference pMCAO model, immunohistochemistry and Western blotting were used to compare cIAP1 protein levels in the five groups on the 3rd, 7th, and 14th days (Figures 7(c) and 7(d)). The results showed that the expression level of cIAP1 in the SA-T and SA-T + Lv *cIAP1* NC groups was upregulated when compared with the model group (*P* < 0.05), while no difference was found between the two groups (*P* > 0.05). The expression level of cIAP1 protein in the SA-T + Lv *cIAP1* group was significantly lower than that in the SA-T group (*P* < 0.05), but there was no difference between the SA-T + Lv *cIAP1* and model groups (*P* > 0.05). This further indicates that SA-T mainly plays a neuroprotective role by targeting cIAP1.

### 3.10. Effect of SA-T on the Caspase-8/-9/-3 Activation Pathway in the cIAP1 shRNA Interference pMCAO Model

**Figure 8:**
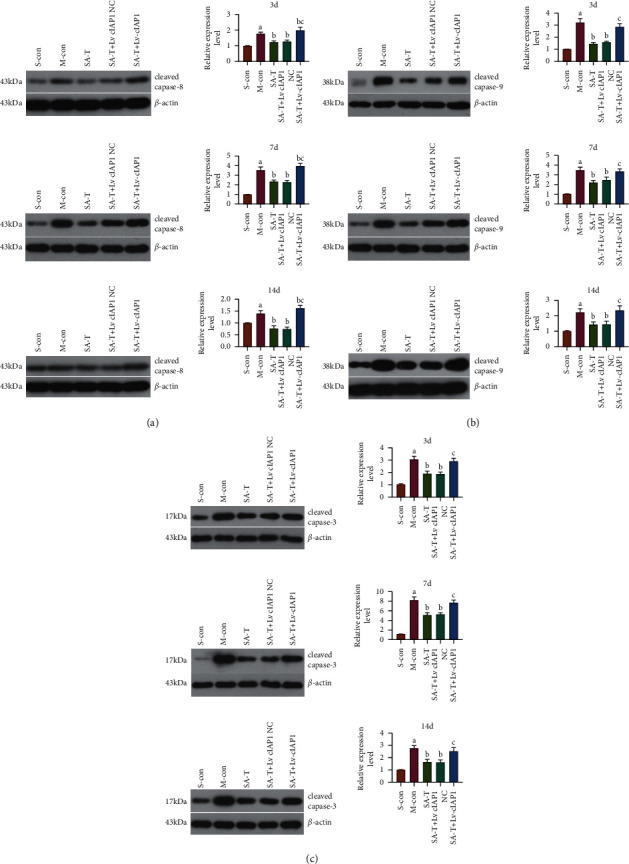
Effect of SA-T on the caspase-8/-9/-3 activation pathway in the cIAP1 shRNA interference pMCAO model. (a) Detection of the expression level of cleaved caspase-8 protein in the brain by Western blot on the 3rd, 7^th^, and 14th days. (b) Detection of the expression level of cleaved caspase-9 protein in the brain by Western blot on the 3rd, 7^th^, and 14th days. (c) Detection of the expression level of cleaved caspase-3 protein in the brain by Western blot on the 3rd, 7^th^, and 14th days. Data are presented as mean ± SEM; *n* = 6 per group at each time point. ^a^*P* < 0.05 vs. S-con group, ^b^*P* < 0.05 vs. M-con group, ^c^*P* < 0.05 vs. SA-T group.

Caspases are synthesized as proenzymes, which are activated by the cleavage of the prodomain at a specific aspartic acid cleaving site. As shown in Figure 8, the Western blotting results showed that SA-T significantly attenuated pMCAO-induced caspase-8, caspase-9, and caspase-3 activation (*P* < 0.05), but no additional effect was observed in the combined therapy with Lv *cIAP1* NC. Moreover, we found that SA-T had no significant reversal effect on the activation of caspase-8, caspase-9, and caspase-3 in the Lv *cIAP1*-transfected pMCAO model when compared with the model group (*P* > 0.05), while there were statistically significant increases as compared with the SA-T group (*P* < 0.05).

## 4. Discussion

Multitudinous clinical practice guidelines recommend early exercise after stroke to promote recovery from physical disability [22]. Yang et al. demonstrated that two weeks of early exercise promotes the recovery of motor and spatial memory in rats 24 h after MCAO [23]. Previous studies have shown that exercise could promote angiogenesis, increase microvessel density, improve cerebral blood supply, and, thus, reduce the cerebral infarction volume [24–26]. These possible mechanisms have been used to explain the positive effects of early exercise. In the present study, our results demonstrated that the combination therapy of SA and TT successfully improves the short-term and long-term motor function in pMCAO rats, which is better than using SA or TT alone.

According to the damage pattern of brain neurons, the ischemic area can be divided into the ischemic core area and the ischemic penumbra area. Neurons in the ischemic core area are necrotic immediately after acute ischemia and are rarely rescued. Conversely, neurons in the ischemic penumbra are less damaged and can recover, thus making them the target of various interventions [27]. Moreover, neurons in the ischemic area can be further injured by activating the apoptosis pathway after acute ischemic stroke [28]. Therefore, inhibiting neuronal apoptosis in the ischemic penumbra is a key strategy to potentially improve neurological function recovery after ischemic stroke and improve patient prognosis. Our results also demonstrated that the combination therapy of SA and TT significantly reduces the apoptosis of neurons in the ischemic penumbra, which is better than using SA or TT alone.

**Figure 9:**
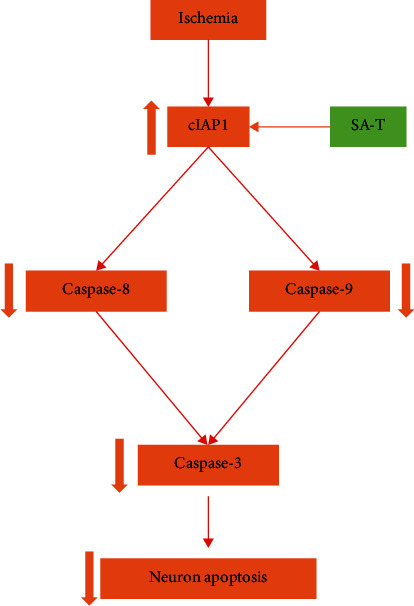
Mechanism diagram. SA-T plays a neuroprotective role through the caspase-independent pathway by upregulating the expression of cIAP1.

Apoptosis, also known as programmed cell death, is a highly regulated mechanism, which mainly activates a series of “caspases” through extrinsic and intrinsic mitochondria pathways to cause nuclear shrinkage, DNA fragmentation, and other apoptosis changes. The caspase-8/-9/-3 activation pathway plays a central role in the execution of apoptosis [29]. The extrinsic pathway, mainly involving the activation of caspase-8 and caspase-10, is initiated by the interaction of cell surface receptors and specific ligands with cell surface receptors such as death receptors, CD95/Fas, and tumor necrosis factor (TNF) [30, 31]. Caspase-9 is involved in the intrinsic mitochondria pathway, which is initiated by various stimuli such as hypoxia and cytotoxic agents, cellular distress, and DNA damage inside the cell [32, 33]. Following the activation of caspase-8 or caspase-9, activated caspase-3 is the final executor of both apoptosis pathways. The activation of caspase-3 results in poly (ADP-ribose) polymerase (PARP) degradation [34]. Previous studies have shown that caspase-8 is a potential target for future therapies, and targeted inhibition of the caspase-8/-3 pathway significantly attenuates the devastating functional losses that result from retinal or cerebral stroke [35]. In the present study, a large number of apoptotic cells were observed in the ischemic penumbra after cerebral ischemia in pMCAO rats when compared with the sham operation group at each time point, and the apoptotic number reached a peak in the acute stage (3 d after ischemia) and gradually decreased in the subacute stage (7 d and 14 d after ischemia). Our results also suggest that SA-T significantly inhibits the activation of the caspase-8/-9/-3 pathway when compared with the model group, so as to play an antiapoptotic role. Therefore, the decrease of neuronal apoptosis mediated by the inactivation of the caspase-8/-9/-3 pathway is an important way for ST-T in the motor function improvement in ischemic stroke (Figure 9).

The inhibitor of apoptosis (IAP) family is involved in various cellular processes, such as cell proliferation, apoptosis, cytokinesis, heavy metal homeostasis, signal transduction, and immunity [36–39]. The IAP family mainly includes cIAP1, cIAP2, X-linked IAP (XIAP), and survivin and is characterized by the presence of at least one baculovirus inhibitor of apoptosis repeat domain that promotes protein-protein interactions. cIAP1 is an important regulator of the apoptosis cascade, which inhibits subsequent exogenous and endogenous apoptosis by blocking caspase activity [40]. Multiple studies have shown that the deficiency of apoptosis caused by the overexpression of cIAP1 is closely related to the occurrence of various tumors and is also related to the poor prognosis of tumors and tumor resistance [41, 42]. Previous studies have confirmed that cIAP1 prevents caspase-3's feed-forward activation of procaspase-9 and can also directly bind to caspase-3 and caspase-7, thus inhibiting their activity [43]. In addition, the degradation of caspase-3 and caspase-7 proteasomes can be mediated by the active ubiquitin dependent on E3 ubiquitin ligase, which is related to the binding of cIAP1 with the IBM structure of activated caspase-3 and caspase-7 through its BIR2 domain [44].

It was also confirmed that the expression of cIAP1 in primary cultured cortical neurons treated with glutamic acid was significantly decreased, and upregulation of cIAP1 could significantly inhibit apoptosis induced by glutamic acid and thus play a neuroprotective role [45]. In contrast, Motomatsu et al. found that hypothermic treatment reduced the levels of TNF-*α* and other inflammatory mediators and proinflammatory cytokines in the rabbit model of transient spinal cord ischemia and inhibited the expression of cIAP1/2 to promote the survival of neurons [46]. Nevertheless, there are a few studies on cIAP1 in ischemic stroke. A study on neurovascular damage after hypoxic-ischemic neonatal brain tissue shows that cIAP1 is a critical endogenous antiapoptotic brake induced by ischemic preconditioning and has a protective effect on neonatal cerebral neurovascular damage [20]. In this study, the expression level of cIAP1 was significantly downregulated in the pMCAO model and negatively correlated with the level of apoptosis when compared with the sham operation group at each time point. SA, TT, and SA-T treatment significantly upregulated the expression level of cIAP1 and inhibited neuronal apoptosis in the ischemic penumbra tissue on the 3rd, 7th, and 14th days after cerebral ischemia.

Herein, our results suggest that the expression level of cIAP1 in the ischemic penumbra tissue is closely related to the apoptosis of neurons. However, the protective effect of SA-T on neurons was not observed in the combined injection of shRNA cIAP1 rats, which further confirmed that the protective effect of SA-T on ischemic neuron injury was mainly dependent on the regulation of cIAP1. Furthermore, the inhibitory effect of SA-T on the caspase-8/-9/-3 activation pathway was completely reversed by intracerebroventricular injection of shRNA cIAP1. These results further confirmed that SA-T regulates the caspase-8/-9/-3 pathway mainly by targeting cIAP1. However, this research has certain time and technical limitations. First of all, this study only explored the neuroprotective mechanism of scalp acupuncture combined with treadmill training therapy from the aspect of nerve apoptosis. Secondly, this study did not conduct in-depth research on the signal transduction pathway of cIAP1 regulating cell apoptosis. Therefore, it has certain limitations. We will further explore from the perspectives of astrocytes and vascular endothelial cells in later studies.

In conclusion, we first report the neuroprotective effect of cIAP1 in ischemic stroke. Mechanistically, cIAP1 inhibits the apoptosis of neurons in the ischemic penumbra by inhibiting the activation of the caspase-8/-9/-3 pathway. Moreover, we found that the combined therapy involving scalp acupuncture and treadmill training improves the motor function by upregulating the expression level of cIAP1, and its short-term and long-term effects are better than scalp acupuncture and treadmill training alone. These results suggest that the combination of scalp acupuncture and treadmill training is a potential rehabilitation strategy for ischemic stroke.

## Data Availability

The data extracted from this Institute's Analytical are stored in the FAIRDOMHub database [47] (https://fairdomhub.org/projects/240).
